# Anti-TIF1-Gamma Autoantibodies-Positive Juvenile Dermatomyositis Associated With Interstitial Lung Disease: A Case Report

**DOI:** 10.7759/cureus.89905

**Published:** 2025-08-12

**Authors:** Ricardo Cid-Puente, Isaac A Carreon-Meza, David A Herrera-VanOostdam

**Affiliations:** 1 Department of Immunology, Biological Sciences School, Universidad Autónoma de Zacatecas, Zacatecas, MEX; 2 Department of Pediatrics, Hospital del Niño y la Mujer “Dr. Alberto López Hermosa”, San Luis Potosi, MEX; 3 Department of Rheumatology, Hospital Central "Dr. Ignacio Morones Prieto", San Luis Potosi, MEX

**Keywords:** anti-tif-1γ, inflammatory myositis, interstitial lung disease, juvenile dermatomyositis, juvenile idiopathic inflammatory myositis

## Abstract

Juvenile dermatomyositis (JDM) is a rare autoimmune disease in children, characterized by muscle inflammation and skin manifestations. This case report describes a nine-year-old boy with JDM associated with anti-TIF1-γ antibodies and interstitial lung disease (ILD). The patient presented with progressive muscle weakness and characteristic skin lesions. Imaging confirmed ILD, and treatment with methylprednisolone pulses and rituximab resulted in significant improvement. This case emphasizes the need for pulmonary monitoring in JDM patients, regardless of antibody profile, and highlights the importance of personalized treatment approaches.

## Introduction

Dermatomyositis (DM) stands as a distinctive autoimmune condition characterized by chronic muscle inflammation and unique skin manifestations. When this condition manifests in children, it takes the form of juvenile dermatomyositis (JDM), representing the most prevalent inflammatory myopathy in childhood [1]. With an incidence rate of 1.9 to 4.1 per million children annually in the USA and UK, JDM predominantly affects young females, typically emerging around age seven [2]. The underlying causes of JDM remain somewhat enigmatic, though current understanding points to a complex interplay between genetic predisposition and environmental factors. Genetically, the condition shows a strong association with specific HLA haplotypes, namely, DQA1*0501 and DRB1*0301 [3]. Environmental triggers play a crucial role, with ultraviolet radiation, certain medications, and various infections, including parvovirus B19, Epstein-Barr virus, coxsackievirus, and mycoplasma, serving as potential catalysts [2,3]. JDM presents primarily with symmetric muscle weakness, predominantly affecting the shoulder and pelvic girdles, and neck flexors, with possible respiratory involvement in severe cases. Characteristic skin manifestations include heliotrope rash, Gottron's papules, shawl and V-signs, and periungual capillary changes. Complications may extend to include non-erosive arthritis, calcinosis, lipodystrophy, vasculopathies, and pulmonary or gastrointestinal involvement [4,5].

The diagnostic landscape for JDM has evolved significantly over time, moving from criteria based on expert consensus to a data-driven, probability-based model. While the 1975 Bohan and Peter criteria, requiring cutaneous lesions plus three additional specific symptoms, served as the traditional diagnostic standard, the field advanced significantly in 2017 with the European League Against Rheumatism (EULAR)/American College of Rheumatology (ACR) classification criteria, which use a weighted scoring system to calculate the probability of myositis. These criteria assign points based on age of onset (<18 for JDM), specific muscle weakness patterns (proximal arm/leg and neck flexor weakness), characteristic skin manifestations (heliotrope rash, Gottron's papules, Gottron's sign), the presence of anti-Jo-1 antibodies, and elevated levels of muscle-associated enzymes (creatine kinase (CK), lactate dehydrogenase (LDH), aspartate transferase (AST), alanine aminotransferase (ALT)). While a muscle biopsy with findings like perifascicular atrophy provides the highest point values, it is no longer mandatory for classification. These newer guidelines offer enhanced sensitivity and specificity, with a cumulative score of 7.5 or higher (when a rash is present) indicating a 90% probability of JDM, allowing for a confident diagnosis in many cases without invasive procedures [6,7].

JDM treatment requires an aggressive approach to prevent long-term complications. First-line therapy consists of high-dose steroids, either as intravenous methylprednisolone pulses or oral prednisone. When steroid-related concerns arise, immunosuppressive agents like methotrexate, mycophenolate mofetil, azathioprine, or cyclophosphamide are employed. Biological therapies, such as rituximab, infliximab, and tofacitinib, have emerged as promising options, though their efficacy requires further validation [1,8,9].

A major breakthrough in understanding JDM came with the discovery of myositis-specific antibodies (MSA) and myositis-associated antibodies (MAA), which have revolutionized both diagnosis and treatment approaches. While MAAs typically appear in patients with overlapping autoimmune conditions, MSAs are detectable in 60-80% of JDM cases and correlate with specific clinical presentations [5,10]. Among these, anti-MDA5 and anti-TIF1 antibodies are particularly significant. Anti-MDA5 antibodies strongly correlate with rapidly progressive interstitial lung disease (ILD), a major source of morbidity and mortality. Conversely, anti-TIF1γ antibodies typically indicate pronounced skin involvement and, in adult patients, potential malignancy, though their association with ILD has been rarely documented [11-13].

In this case report, we document a rare and clinically significant presentation of JDM in a nine-year-old child who developed the condition with positive anti-TIF1-γ antibodies and concurrent ILD. Through detailed examination of this case, we aim to contribute to the growing body of knowledge regarding atypical presentations of JDM and their management, potentially influencing future diagnostic and treatment strategies for similar cases.

## Case presentation

**Figure 1:**
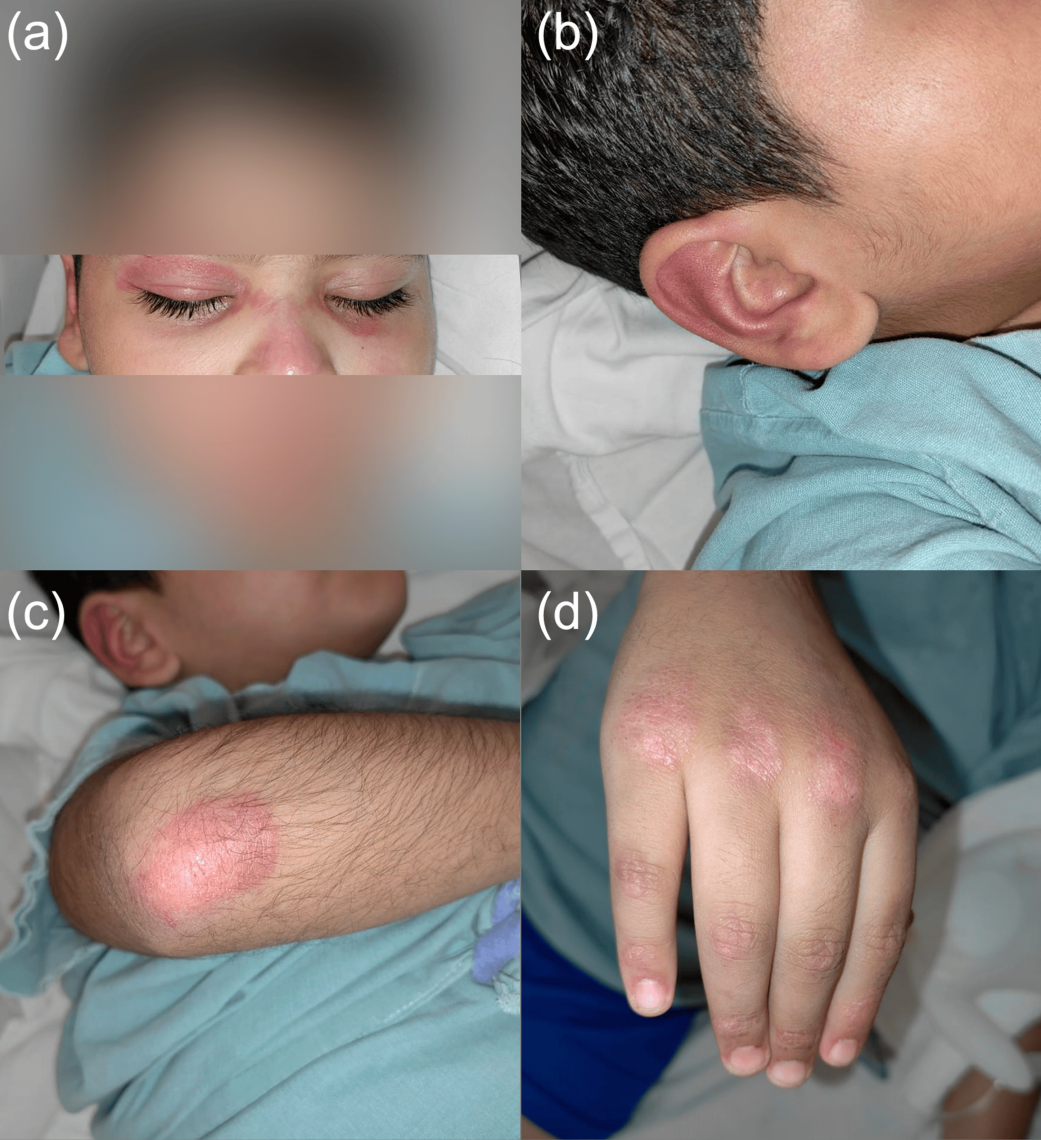
Pathognomonic juvenile dermatomyositis skin lesions (a) Heliotrope rash in both eyelids. (b,c) Erythemato-violaceous plaques on the right ear and elbow. (d) Gottron's papules on the knuckles.

A previously healthy nine-year-old boy with no significant medical history presented to our hospital with an eight-month history of progressive weakness in all four extremities. The weakness had worsened over the past three months, limiting his ability to walk more than one block, run, or jump, and had resulted in multiple falls. He also exhibited erythematous plaques on his face and knuckles. Upon admission to the pediatric department, he was conscious and oriented with normal vital signs and mild dyspnea. Physical examination revealed proximal muscle weakness (69/150 on the Manual Muscle Testing 8 (MMT8) scale), heliotrope rash, Gottron's papules, and pruritic erythematous-violaceous plaques on the ears, right elbow, and anterior aspect of the right leg (Figure 1). Nailfold capillary changes, including increased capillary density and megacapillaries, were observed.

**Figure 2:**
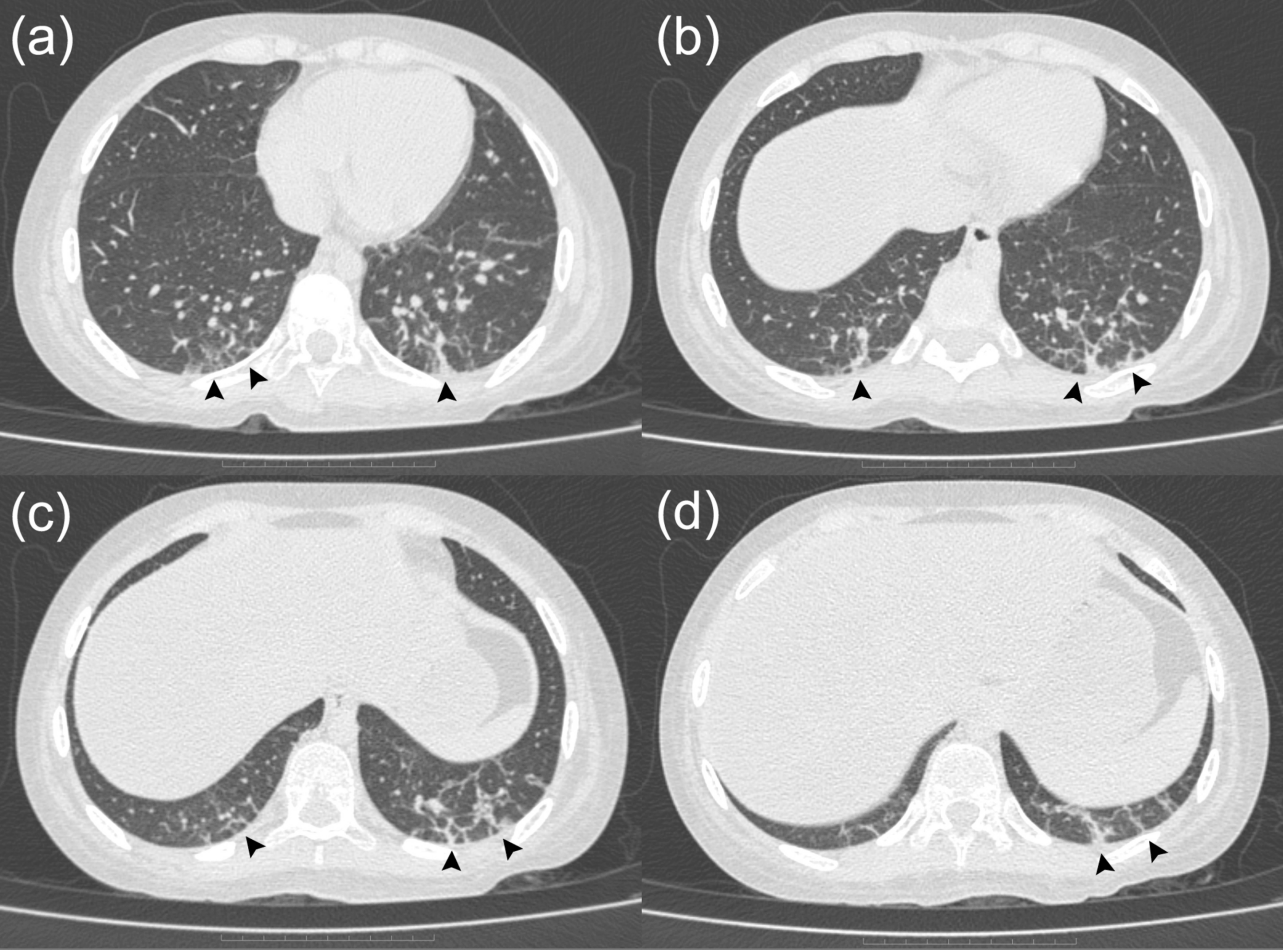
Axial non-contrast computed tomography of the lungs Black arrowheads indicate fibrosis: (a, b) middle pulmonary lobes; (c, d) lower pulmonary lobes

Laboratory tests showed normal muscle enzyme levels. Antinuclear antibodies were positive at a lower titer. Anti-TIF1-γ antibodies were strongly positive, while anti-Jo1, anti-Mi2, anti-MDA5, and anti-NXP2 antibodies were negative (Table 1). Electromyography (EMG) revealed an inflammatory myopathic pattern, and high-resolution computed tomography of the chest was requested due to dyspnea, demonstrating evidence of ILD in both lung bases (Figure 2).

**Table 1:** Laboratory findings

Laboratory tests	Result	Reference range
Hemoglobin (g/dL)	14.4	14.0-16.0
White blood count (1,000/uL)	6.4	4.5-11.0
Platelets (1,000/uL)	314.0	150.0-450.0
Aspartate aminotransferase (UI/L)	47.0	17.0-59.0
Alanine aminotransferase (UI/L)	28.0	0.0-50.0
Erythrocyte sedimentation rate (mm/hr)	7.0	<15mm/hr
Creatine phosphokinase (UI/L)	78.0	0.0-20.0
Antinuclear antibodies	1:80	Negative
Anti-TIF1- γ	Positive	Negative
Anti-Jo-1	Negative	Negative
Anti-Mi2	Negative	Negative
Anti-MDA5	Negative	Negative
Anti-NXP2	Negative	Negative

Based on the comprehensive clinical presentation, immunological profile, and EMG findings, a definitive diagnosis of JDM was established. The patient scored 13.6 points on the EULAR/ACR classification criteria, corresponding to an estimated 99% probability of JDM, well exceeding the 7.5-point threshold required for confident diagnosis.

The patient was treated with high-dose methylprednisolone pulses (24 mg/kg/day for 3 days) and a single dose of intravenous rituximab (500 mg). Five days after initiating treatment, there was a noticeable improvement in cutaneous lesions and a slight improvement in limb strength. The patient was discharged after one week with a treatment regimen of prednisone (50 mg daily), methotrexate (12.5 mg weekly), mycophenolate mofetil (500 mg every 12 hours), and folic acid (5 mg daily). Follow-up appointments were scheduled at the pediatric rheumatology clinic to monitor disease activity and adjust treatment as necessary.

## Discussion

The relationship between anti-TIF1-γ antibodies and ILD in JDM presents an intriguing clinical paradox that warrants careful consideration. Anti-TIF1γ antibodies stand as one of the most frequently detected MSAs, appearing in a substantial 16-35% of JDM cases. However, what makes this case particularly exceptional is the strikingly low association of these antibodies with ILD - a mere 6.1% in pediatric cases and 8.7% in adults [14,15]. This remarkably low incidence becomes even more intriguing when examining the underlying molecular mechanisms. The TIF1γ protein, the target of these antibodies, demonstrates inherent protective properties against pulmonary fibrosis, which likely explains the unusually low prevalence of ILD in this patient population [15]. These statistics and biological mechanisms underscore just how extraordinary it is to encounter a case where anti-TIF1γ antibodies and ILD coexist, making our reported case an important contribution to the medical literature and our understanding of potential disease variations.

The disparity between adult and pediatric presentations adds another layer of complexity to our understanding. In adult DM, anti-TIF1-γ antibodies strongly correlate with malignancy, and the elevated TIF1-γ protein levels observed in cancer patients may contribute to their protection against ILD [16]. However, this protective mechanism cannot fully explain the low ILD prevalence in children, where malignancy is exceedingly rare [17]. Our analysis suggests that several factors may contribute to this phenomenon. First, children with anti-TIF1-γ-positive JDM might develop subclinical ILD that goes undetected due to limited routine imaging in asymptomatic patients. Additionally, the aggressive immunosuppressive treatment typically initiated early in JDM may prevent the progression to clinically significant ILD.

The contrast with anti-MDA5-positive patients, where up to 79% develop rapidly progressive ILD, highlights the distinct pathogenic mechanisms associated with different autoantibody profiles [18]. This difference in ILD risk has important implications for clinical management, suggesting that while anti-MDA5-positive patients require vigilant monitoring for lung involvement, anti-TIF1-γ-positive patients might benefit more from a focus on their characteristic features such as severe cutaneous manifestations and chronic disease course. However, this should not lead to complacency regarding potential lung involvement, as some anti-TIF1-γ-positive patients do develop ILD, albeit less frequently and usually less severely.

Furthermore, the observation that anti-TIF1γ-positive patients often exhibit more severe cutaneous disease but milder systemic manifestations points to the tissue-specific effects of these autoantibodies. The clinical implications of these findings are significant for patient care. While the risk of ILD in anti-TIF1-γ-positive JDM patients appears lower, the potential for subclinical disease progression suggests the need for periodic pulmonary assessment, even in asymptomatic patients. The development of biomarkers for early ILD detection and disease progression monitoring could significantly improve patient outcomes. Additionally, understanding the protective mechanisms of TIF1-γ against pulmonary fibrosis could lead to novel therapeutic strategies not only for JDM but also for other fibrotic lung diseases.

Moving forward, several key questions require further investigation. Longitudinal studies are needed to better understand the natural history of subclinical ILD in anti-TIF1-γ-positive JDM patients. The molecular mechanisms underlying TIF1-γ's protective effects against pulmonary fibrosis deserve detailed examination, as they might reveal new therapeutic targets.

Finally, the development of standardized protocols for pulmonary screening in JDM patients, considering their autoantibody status, could help optimize resource utilization while ensuring appropriate monitoring of at-risk patients. This comprehensive approach to understanding the relationship between anti-TIF1-γ antibodies and ILD in JDM has the potential to significantly improve patient care through more targeted and evidence-based management strategies.

## Conclusions

This case report highlights the unique presentation of JDM with positive anti-TIF1-γ antibodies and ILD, challenging our current understanding of the relationship between these antibodies and pulmonary involvement. While anti-TIF1-γ antibodies are typically associated with severe cutaneous manifestations and lower ILD risk, this case demonstrates that pulmonary involvement can occur in these patients. This case emphasizes the need for regular pulmonary screening in all JDM patients, regardless of their antibody profile, and suggests that our understanding of the protective role of TIF1-γ against pulmonary fibrosis requires further investigation. Moving forward, the development of standardized screening protocols and continued research into the molecular mechanisms of TIF1-γ could lead to more targeted therapeutic approaches and improved patient outcomes in JDM management.
